# Coronary Microvascular Dysfunction: What Clinicians and Investigators Should Know

**DOI:** 10.1007/s11883-023-01116-z

**Published:** 2023-06-20

**Authors:** Paul Marano, Janet Wei, C. Noel Bairey Merz

**Affiliations:** 1 Cedars-Sinai Medical Center, Smidt Heart Institute, Los Angeles, CA USA; 2 Cedars-Sinai Medical Center, Barbra Streisand Women’s Heart Center, Smidt Heart Institute, 127 S. San Vicente Blvd, Los Angeles, CA 90048 USA

**Keywords:** Coronary microvascular dysfunction, Microvascular angina, Coronary function testing, Coronary flow reserve, Ischemia with no obstructive coronary artery disease

## Abstract

**Purpose of Review:**

Abnormal structure and function of the coronary microvasculature have been implicated in the pathophysiology of multiple cardiovascular disease processes. This article reviews recent research progress related to coronary microvascular dysfunction (CMD) and salient clinical takeaways.

**Recent Findings:**

CMD is prevalent in patients with signs and symptoms of ischemia and no obstructive epicardial coronary artery disease (INOCA), particularly in women. CMD is associated with adverse outcomes, including most frequently the development of heart failure with preserved ejection fraction. It is also associated with adverse outcomes in patient populations including hypertrophic cardiomyopathy, dilated cardiomyopathy, and acute coronary syndromes. In patients with INOCA, stratified medical therapy guided by invasive coronary function testing to define the subtype of CMD leads to improved symptoms.

**Summary:**

There are invasive and non-invasive methodologies to diagnose CMD that provide prognostic information and mechanistic information to direct treatment. Available treatments improve symptoms and myocardial blood flow; ongoing investigations aim to develop therapy to improve adverse outcomes related to CMD.

## Introduction 

Coronary microvascular dysfunction (CMD) describes abnormalities in the structure and function of the coronary microcirculation that occur across a variety of cardiovascular conditions. While the diagnostic and therapeutic focus in patients with suspected ischemic heart disease has traditionally been on obstructive atherosclerosis in the epicardial coronary arteries, there is now greater appreciation of the impact of structural and functional disorders affecting the entire coronary circulation, including the microcirculation. CMD is an increasingly recognized cause of angina, and has prognostic importance in multiple cardiovascular disease processes, including its association with adverse outcomes in patients with signs and symptoms of ischemia but no obstructive coronary arteries (INOCA). In this review, we will summarize what is known about the pathophysiology of CMD and discuss clinical presentations, strategies for diagnosis, and the current treatment of CMD, focusing on practical information for the clinical care of patients with CMD.

## Pathophysiology

The coronary arterial system can be conceptually divided into three compartments with progressively decreasing diameter and distinct physiology [1]. The most proximal compartment is comprised of the epicardial coronary arteries (0.5 – 5.0 mm in diameter), which function as capacitance vessels and, in the absence of obstructive stenoses, do not contribute significant resistance to coronary blood flow. After the epicardial arteries, the remaining two compartments, the pre-arterioles (0.1 – 0.5 mm in diameter) and intramyocardial arterioles (< 0.1 mm in diameter), which interface directly with the capillary bed, together make up the coronary microcirculation. Notably, the epicardial arteries represent only 10% of the coronary circulation volume, while the microcirculation contains the remaining 90% [2]. The microcirculation is the site of the majority of the resistance to coronary blood flow and its regulation [3].

The dynamic regulation of microcirculatory resistance maintains coronary blood flow across a wide range of perfusion pressures and matches blood flow to myocardial oxygen consumption. Because the oxygen extraction of myocardial cells is near-maximal under physiologic conditions, the changing metabolic demands of the myocardium must be met by changes in coronary blood flow [4]. In health, these dynamic changes in coronary blood flow occur by the regulation of arteriolar tone through several interconnected pathways [5]. Increased myocardial oxygen consumption leads to the local release of metabolites that trigger dilation of the small intramyocardial arterioles. In the pre-arterioles, myogenic and shear stress-induced mechanisms control resistance, along with input from the adrenergic nervous system [6]. These interconnected regulatory pathways in the microcirculation work together to titrate coronary blood flow to match myocardial oxygen requirements; an increase in myocardial oxygen demand is met by a proportional increase in coronary blood flow. In CMD, these regulatory pathways are disrupted through a combination of structural and functional abnormalities, which can result in ischemia and symptoms.

Structural factors implicated in CMD include decreased capillary density, luminal narrowing of arterioles/capillaries related to edematous endothelial cells and proliferated smooth muscle cells, and external compression [7–9].

Functional mechanisms include impaired endothelium-dependent dilation, impaired endothelium-independent dilation, and enhanced constrictive reactivity. Endothelial cells in both the epicardial arteries and pre-arterioles respond to shear stress created by changes in blood flow. Endothelial dysfunction leads to a blunted response to typical triggers for microvascular dilation, such as exercise or exposure to acetylcholine [10, 11]. Furthermore, endothelial dysfunction can even lead to a vasoconstrictive response rather than a blunted vasodilatory response to these triggers, and is therefore implicated in vasospasm [12]. Aside from the endothelium, the myogenic response of the microvasculature is abnormal in CMD; this can be observed in the attenuated response to vasodilators such as adenosine that targets vascular smooth muscle cells directly.

## Definitions

Historically, inconsistent definitions and unclear terminology reflected our limited understanding of the prevalence and pathophysiology of CMD. The nonspecific term “cardiac syndrome X” was previously used to refer to patients with chest pain despite apparently normal angiograms, with or without objective evidence of ischemia or CMD. This term encompassed a heterogenous group of patients with various cardiac and noncardiac etiologies for chest pain [13]. Recent efforts have standardized definitions of CMD and the entity of chest pain attributable to CMD, microvascular angina. The term cardiac syndrome X is no longer used. CMD is now defined by specific functional tests designed to assess the microvascular response to vasoactive medications, as we will review later.

The Coronary Vasomotor Disorders International Study Group (COVADIS) set out diagnostic criteria for microvascular angina and vasospastic angina [14, 15]. The criteria for microvascular angina included (1) symptoms of myocardial ischemia; (2) absence of obstructive coronary artery disease (CAD); (3) objective evidence of myocardial ischemia; and (4) evidence of impaired coronary microvascular function.

## Clinical Presentation

Patients with CMD present with a spectrum of symptoms, similar to patients with obstructive epicardial CAD, including typical angina pectoris, atypical chest pain, and angina-equivalent symptoms, such as dyspnea on exertion. Compared to patients with angina due to obstructive epicardial CAD, patients with microvascular angina tend to have less therapeutic response to nitroglycerin [16]. On initial evaluation, these patients frequently have electrocardiographic evidence of ischemia on ECG stress testing, though may or may not have evidence of hypoperfusion on traditional myocardial perfusion imaging [17]. Any observed perfusion defect may not be in a typical regional distribution corresponding to an epicardial artery, and contractile abnormalities are typically not observed on stress echocardiography. The difficulty in observing ischemia by traditional imaging methods may be related to a nonuniform distribution of dysfunctional microvasculature [17].

Most commonly, patients with CMD present with anginal symptoms and signs as above, undergo an evaluation for obstructive CAD and are found to have non-obstructive atherosclerosis. While this is most common, there are variable presentations of CMD, as multiple cardiovascular disease processes affect the microvasculature. In 2007, Camici and Crea classified CMD into distinct types based on the presence or absence of obstructive epicardial CAD and myocardial disease [1]. This framework was modified by Taqueti and Di Carli in 2018 to address the overlap between multiple disease states involving CMD and to emphasize the close relationship of CMD with atherosclerosis [18]. According to this model, CMD can be separated into three categories: CMD with non-obstructive epicardial CAD, CMD with obstructive epicardial CAD, and CMD unrelated to atherosclerosis.

### CMD with Non-Obstructive Epicardial Coronary Artery Disease

We will first discuss the most prevalent presentation of CMD. A substantial number of patients with clinical evidence of ischemia who undergo coronary angiography for suspected ischemia do not have obstructive epicardial CAD (INOCA). Estimates from the WISE study suggest that 3–4 million patients in the USA fit this description [19]. Similarly, in a large registry of approximately 400,000 patients referred for coronary angiography, only 41% of patients with a positive non-invasive test had obstructive CAD [20]. CMD has been implicated as a common cause of ischemia and symptoms in these patients. In both the original WISE study (1997–2001) and the WISE-CVD study (2009–2012), nearly half of women with symptoms of ischemia but no obstructive CAD had CMD detected by invasive testing [21, 22•], which has subsequently been replicated in additional populations [23, 24]. While there is no obstructive CAD, the majority of these patients do have evidence of atherosclerosis, suggesting a link in the pathophysiology of CMD and atherosclerosis [25].

It follows that conditions associated with increased risk of CMD include traditional atherosclerosis risk factors such has age [26], hypertension [27], diabetes [28, 29], dyslipidemia [30], and chronic kidney disease [31]. In addition, there is evidence supporting an association between inflammation and CMD; inflammatory markers such as C-reactive protein are inversely correlated with microvascular function [32, 33], and patients with inflammatory disorders such as psoriasis have high rates of CMD [34], which improves with anti-inflammatory therapy [35]. Chronic inflammation has a role in the pathogenesis of CMD, as it does in the development and progression of atherosclerosis [36, 37]. However, traditional CAD risk factors do not fully explain the risk of CMD; these risk factors only explained < 20% of the observed variability in the coronary reactivity response to adenosine in the WISE cohort [38, 39]. Further work is needed to understand the conditions that contribute to CMD in the presence of non-obstructive atherosclerosis.

While CMD is prevalent in both men and women presenting for evaluation of chest pain with INOCA [40], studies have observed relatively higher rates in women [41]. Importantly, CMD is a major driver of adverse outcomes including cardiovascular death and hospitalization for myocardial infarction or heart failure in this population, particularly in women [42•, 43]. CMD is therefore an important factor in understanding the observation of similar or worse outcomes of atherosclerosis in women despite a lower rate of obstructive epicardial CAD [42•, 44].

There is a growing body of evidence supporting a role for CMD in the pathogenesis of heart failure with preserved ejection fraction [45–47], and presentations of dyspnea in patients with CMD may represent symptomatic heart failure. Additional populations in which CMD plays an important role are heart transplant patients with coronary allograft vasculopathy [48] and Takotsubo syndrome [49, 50].

### CMD with Obstructive Epicardial Coronary Artery Disease

CMD can also co-occur with obstructive epicardial CAD, in both chronic and acute coronary syndromes. In chronic coronary syndromes, there is indirect evidence of the importance of the microcirculation from recent trials in which percutaneous coronary intervention on the epicardial coronary arteries did not improve adverse outcomes compared to medical therapy (ISCHEMIA) [51] and did not improve symptoms compared to sham control (ORBITA) [52].

It is also important to understand the potential impact of the microcirculation when assessing the hemodynamic significance of epicardial stenoses. Invasive measures of the physiologic significance of epicardial stenoses, such as the fractional flow reserve and the instantaneous wave-free ratio, are affected by the presence of CMD. These measurements are higher in the presence of CMD, which can lead to underestimation of the hemodynamic significance of epicardial lesions [53].

In acute coronary syndromes, both before and after percutaneous intervention, microvascular obstruction can occur related to multiple mechanisms including distal embolization of plaque/thrombi, the release of vasoactive substances, and compression due to myocardial edema or hemorrhage [54]. Microvascular obstruction can be detected angiographically (no-reflow phenomenon, abnormal myocardial blush), by incomplete ST-segment resolution despite successful revascularization, or by various imaging techniques in the days after myocardial infarction and/or intervention. As assessed by cardiac magnetic resonance imaging, approximately half of patients with ST-elevation myocardial infarction treated with percutaneous intervention have evidence of microvascular obstruction, with important implications for long-term outcomes [55].

### CMD Unrelated to Atherosclerosis

CMD can also occur in a variety of cardiovascular diseases in which atherosclerosis is not present, and has an important pathophysiologic and prognostic role in these conditions. Among these, CMD has been identified in hypertrophic cardiomyopathy, where structural changes to the microvasculature including medial hypertrophy, intimal hyperplasia, and decreased luminal size have been observed [56]. In hypertrophic cardiomyopathy, abnormalities in the microvasculature are associated with increased fibrosis [56]. It has been proposed that ischemia related to CMD is an important driver of fibrosis and adverse outcomes in these patients [57].

CMD has been identified in infiltrative cardiomyopathies including cardiac amyloidosis and Fabry disease [58, 59]. In aortic stenosis, CMD and hemodynamic disturbances lead to reduced myocardial blood flow and adverse outcomes [60]. Previous studies have also identified CMD in dilated cardiomyopathy, and the degree of CMD has been shown to predict adverse outcomes [61].

### Clinical Recognition of CMD

It is important for the clinician to recognize that CMD can be a source of anginal symptoms and ischemia in patients within each of these classifications. A patient with ischemia on myocardial perfusion imaging and diffuse non-obstructive epicardial CAD on angiography, a patient with chest pain after stenting but without in-stent thrombosis or other epicardial complications, and a patient with hypertrophic cardiomyopathy with exertional chest pain may all have ischemia and pain related to CMD.

## Diagnosis

The diagnosis of CMD should be suspected, and further testing considered when there are symptoms of angina and/or objective signs of ischemia on non-invasive testing without explanatory obstructive epicardial coronary artery disease. For the clinician, this represents a frame shift away from a “plaque-centric” model in which the goal is to identify and treat obstructive epicardial plaques. In this prior conception, evidence of ischemia with no obstructive epicardial plaques was often considered a false positive and patients were reassured. With a large body of evidence now supporting an elevated risk of adverse outcomes in patients with CMD, further diagnostic testing to evaluate for CMD is often warranted.

**Fig. 1:**
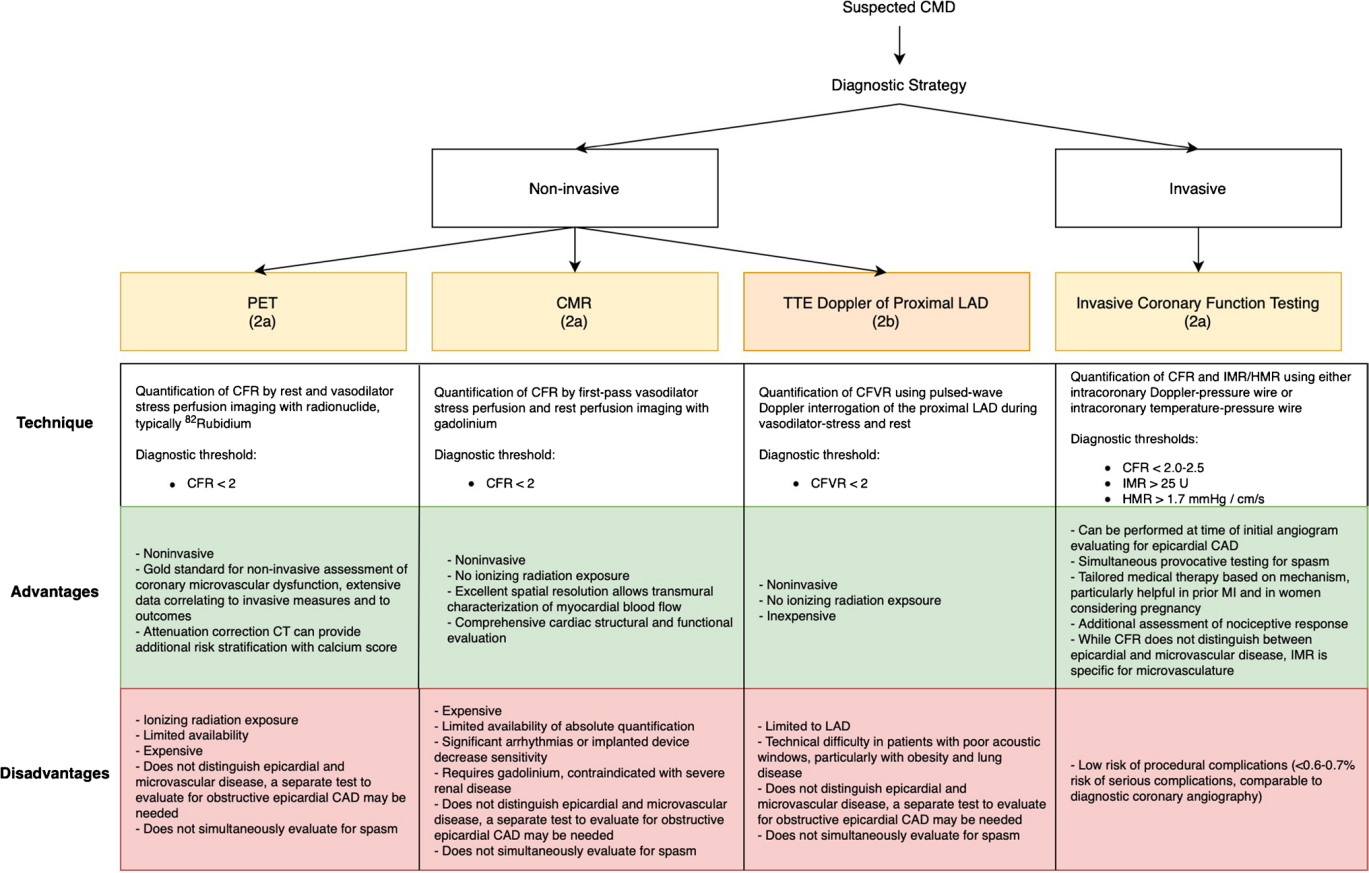
Diagnostic strategy for suspected coronary microvascular dysfunction. When coronary microvascular dysfunction (CMD) is suspected based on signs and/or symptoms of ischemia in the absence of explanatory obstructive epicardial coronary artery disease (CAD), the following non-invasive and invasive methodologies are recommended for further evaluation. Measurement of coronary flow reserve (CFR) by positron emission tomography (PET) and cardiac magnetic resonance imaging (CMR) received a 2a recommendation in the recent AHA/ACC 2021 Chest Pain Guidelines [62], while stress echocardiography (TTE) with Doppler assessment of left anterior descending artery (LAD) flow velocity reserve received a 2b recommendation for patients with persistent stable chest pain and nonobstructive CAD. CFR or coronary flow velocity reserve values below 2 suggest CMD. For those patients that also have at least mild ischemia on imaging, invasive testing was given a 2a recommendation. On invasive coronary function testing, in addition to CFR, the index of myocardial resistance (iMR) can be calculated based on the distal pressure measured by the intracoronary wire and the transit time of the saline bolus between the proximal and distal temperature sensors. This is a measure that specifically targets the microcirculation (the hyperemic microvascular resistance, or HMR is an analogous calculation with measurements obtained using a Doppler-pressure intracoronary wire). Advantages and disadvantages of the different modalities are discussed

The coronary microcirculation is too small to be directly visualized in vivo. It must be assessed functionally through either invasive or non-invasive testing. In the 2021 AHA/ACC/ASE/CHEST/SAEM/SCCT/SCMR Guideline for the Evaluation and Diagnosis of Chest Pain, both invasive coronary function testing and non-invasive assessment of myocardial blood flow by positron emission tomography (PET) or stress cardiac magnetic resonance (CMR) imaging were given class 2a recommendations for patients with persistent stable chest pain and non-obstructive coronary artery disease [62] (Fig. 1). The European Society of Cardiology Chronic Coronary Syndrome guidelines from 2019 provide similar recommendations for testing [63].

### Invasive coronary function testing

The most comprehensive diagnostic assessment for CMD is invasive coronary function testing. This procedure, performed in the catheterization lab, allows for assessment of the macrovascular and microvascular response to multiple vasoactive agents, both through endothelium-dependent and endothelium-independent mechanisms [23]. Prior to functional testing, coronary angiography is used to exclude previously undiagnosed obstructive epicardial CAD, significant myocardial bridging, coronary anomalies, or other abnormalities.

Adenosine, a vasodilator acting on smooth muscle cells, is administered to assess the coronary flow reserve (CFR). The CFR is calculated as the ratio of hyperemic myocardial blood flow to rest myocardial flow. It is a measure of coronary vasomotor function, integrating the hemodynamic effects across the entire coronary circulation. CMD can therefore be defined as reduced CFR in the absence of obstructive epicardial stenoses. During invasive coronary function testing, the CFR is calculated using a Doppler-tipped guidewire or thermodilution techniques to measure myocardial blood velocity/flow at rest and after induction of hyperemia with adenosine. Since adenosine acts directly on smooth muscle cells, an abnormal CFR with no obstructive CAD reflects endothelium-independent microvascular dysfunction.

Another invasive measure that reflects the endothelium-independent microvascular function is the index of microvascular resistance (IMR). This calculation requires simultaneous use of a pressure guidewire and thermodilution techniques, and is calculated as the distal coronary pressure at maximal hyperemia multiplied by the hyperemic mean transit time. An IMR $\ge$ 25 is abnormal and consistent with CMD. While CFR reflects the entire coronary circulation from the epicardial arteries to the microcirculation, the IMR is specific to the microcirculation. Compared with CFR measurements, IMR is more reproducible and is less dependent on hemodynamic changes [64, 65]. Recent work indicates improved reproducibility with the use of continuous rather than bolus thermodilution to measure CMD [66].

In addition to assessing the endothelium-independent coronary microvascular function through these hyperemic measurements, invasive coronary function testing can assess the endothelium-dependent microvascular and macrovascular function and assess for spasm. This is achieved through the administration of intracoronary acetylcholine. The macrovascular endothelium-dependent function is assessed by quantitative coronary angiography; epicardial vasodilation is expected in response to acetylcholine, and dilation ≤ 5% suggests dysfunction. For the microvascular endothelium-dependent function, the change in the coronary blood flow is measured in response to acetylcholine, with an increase in flow < 50% suggesting dysfunction. Macrovascular vasospasm can also be detected by angiography in response to acetylcholine, with a > 90% decrease in coronary artery diameter defining spasm. Microvascular spasm can be detected by chest pain, and ischemic ECG changes in response to acetylcholine, with no evidence of epicardial spasm on angiography. Throughout the procedure, pain with catheter manipulation and/or contrast administration is suggestive of a nociceptive abnormality.

Coronary function testing has been shown to be a safe procedure with a low rate of complications comparable to diagnostic coronary angiography [67]. Intracoronary acetylcholine administration has also been demonstrated to be a safe procedure. The rate of serious adverse events, which include coronary artery dissection, arrhythmia, and myocardial infarction, was 0.5% in one large series [68].

As compared with non-invasive techniques, invasive coronary function testing provides additional stratification of the mechanism of the abnormality, which may allow for more directed treatment (Fig. 1). Macrovascular and microvascular spasm, as well as nociceptive abnormality, can be detected by invasive coronary function testing but not by the non-invasive methodologies to assess for CMD. In a meta-analysis of studies assessing the prevalence of CMD and vasospastic angina in patients with no obstructive CAD, epicardial vasospasm was identified in 40% of patients [69]; in these cases, identification of spasm by invasive coronary function testing would have important implications for treatment. This is particularly salient in patients with a prior history of myocardial infarction with no obstructive coronary artery disease (MINOCA), for which vasospasm is a possible cause. Furthermore, invasive coronary function testing often diagnoses coronary endothelial dysfunction in patients with INOCA without inducible spasm and normal adenosine CFR [70•], providing therapeutic implications.

### Non-Invasive Testing

The diagnosis of CMD requires demonstration of impaired myocardial blood flow during hyperemia in the absence of obstructive epicardial CAD. In addition to invasive testing, these parameters can be quantified by non-invasive methodologies including positron emission tomography (PET), cardiac magnetic resonance imaging (CMR), and transthoracic Doppler echocardiography of the left anterior descending artery (LAD) (Fig. 1).

PET is the most studied non-invasive technique for the diagnosis of CMD. The measurements required to assess for CMD are incorporated into typical protocols for PET myocardial perfusion stress testing. Images are obtained following the injection of a radionuclide (typically ^82^Rubidium or ^13^N-ammonia in the US) at rest and with vasodilator stress. The characteristics of PET imaging allow for the absolute quantification of global and regional myocardial blood flow (ml/min/g) by measuring the total tracer activity delivered to the myocardium as a function of time [71]. The CFR can be calculated by the ratio of the myocardial blood flow at stress to rest, and a CFR < 2 in the absence of epicardial CAD is consistent with CMD. The accuracy and reproducibility of CFR calculated by PET are well-established [72]. Disadvantages of PET include the ionizing radiation and lack of availability at many centers.

Another noninvasive method to assess for CMD is stress CMR [73–75]. Semiquantitative and quantitative models of rest and vasodilator stress first-pass myocardial perfusion of gadolinium allow for calculation of myocardial perfusion reserve. Advantages of CMR relative to PET include availability in more centers, high spatial resolution permitting characterization of the transmural extent of perfusion, and the lack of ionizing radiation.

Finally, transthoracic Doppler echocardiography of the proximal LAD can be used to calculate the coronary flow velocity reserve, which is calculated as the ratio of the coronary flow velocity at stress and at rest obtained by using a pulse-wave Doppler interrogation of the proximal LAD [76–78].

## Treatment

The treatment of CMD is aimed at reducing the risk of adverse events and improving symptoms. Existing management guidelines in the USA do not provide specific recommendations for treatment, as there is not robust evidence from large-scale randomized trials to support therapy. The most recent 2019 European guidelines on Chronic Coronary Syndromes support treatment directed at the dominant mechanism of microcirculatory dysfunction based on the available trial evidence; treatment is currently based on reducing the risk of adverse cardiovascular outcomes related to the presence of associated conditions such as atherosclerosis or heart failure, and treating symptoms targeted to subtype of CMD (Table 1).

**Table 1:** Selected studies of pharmacologic and non-pharmacologic treatment of coronary microvascular dysfunction

Treatment	Evidence of benefit in CMD
*Pharmacologic therapy*	
*Targeted at atherosclerosis*	
Aspirin	• Extrapolated from benefit in atherosclerotic disease
Statin	• **Improved endothelium-dependent function** (assessed invasively) in 25 patients with treated hypercholesterolemia compared to untreated [80] • **Improved CFR** (assessed by TTE Doppler) in 20 patients after 8 weeks, no control [100]
ACEi/ARB	• **Improved CFR (invasive) and exercise duration** in 10 patients randomized to ACEi compared to placebo after 8 weeks [81] • **Improved CFR and anginal symptoms** in 29 women randomized to ACEi compared to placebo at 16 weeks [82]
*Anti-anginal*	
Beta-blockers	• **Improved CFR** (by PET) in 25 patients with dilated cardiomyopathy randomized to carvedilol compared to placebo [101] • **Improved CFR** (invasive) after intracoronary nebivolol in 8 patients with CAD and 10 controls [87] • **Improved anginal symptoms** in 10 patients given atenolol compared to nitrate, amlodipine at 4 weeks (crossover design) [102]
Calcium channel blockers	• **Improved epicardial spasm**, no improvement in other measures of CMD (invasive) at 6 weeks in 38 patients with CMD randomized to diltiazem compared to placebo [91] • **Improved exercise duration, improved anginal symptoms** with verapamil or nifedipine in 26 patients with CMD, compared with placebo crossover [103]
Nitrates	• Short acting nitrates did not improve time to ST-depression during stress test in 29 patients with microvascular angina [16]
Ranolazine	• **Improved angina and myocardial perfusion** (by CMR) in 35 women with CFR < 2.5 randomized to ranolazine compared to placebo (crossover) [104]
Ivabradine	• **Improved angina** in 46 patients with CMD compared to placebo (crossover) [105]
*Non-pharmacologic therapy*	
Exercise/weight loss	• **Improved CFR** at 12 weeks in 26 obese patients assigned to aerobic exercise and 24 assigned to low-calorie diet [97]
Smoking cessation	• Comparison of smoking-discordant twin pairs, **lower CFR in smoking** twin vs non-smoking twin [98]
Enhanced external counterpulsation	• **Improved angina** in 30 patients with CMD (no control) [99]

Bolded text highlights the studied outcome in the various trials

*CMD*, coronary microvascular dysfunction; *CFR*, coronary flow reserve; *TTE*, transthoracic echocardiography; *ACEi*, angiotensin-converting enzyme inhibitor; *ARB*, angiotensin receptor blocker; *PET*, positron emission tomography; *CAD*, coronary artery disease; *CMR*, cardiac magnetic resonance imaging

### Treatment Related to Atherosclerosis

As noted, CMD often coexists with some degree of atherosclerosis. In this population, therapies aimed at reducing the risk of major adverse cardiac events in the setting of atherosclerosis are employed. To this end, aspirin and statin therapy are often used. Regarding their specific benefit in CMD, there have been small trials indicating improved CFR and improved exercise-induced ischemia with statin therapy [79, 80] (Table 1).

Similarly, angiotensin-converting enzyme inhibitors (ACEi) reduce adverse events and are recommended in patients with chronic coronary syndromes and concurrent conditions including heart failure, hypertension, or diabetes [63]. These medications are therefore extended to patients with CMD, particularly the high number of patients with these coexisting conditions. There is evidence from multiple small trials supporting improved CFR after treatment with ACEi, exercise time, and symptoms in patients with CMD [81, 82]. For patients who are intolerant to ACEi, angiotensin receptor blockers (ARB) also have been shown to improve CFR, although were inferior to ACEi in a small trial [83–85].

The forthcoming Women’s Ischemia Trial to Reduce Events in Non-Obstructive CAD (WARRIOR) trial will provide important data regarding the impact of medical therapy on intermediate-term outcomes [86••]. The WARRIOR trial will test the impact at 3-year follow-up of intensive medical therapy including aspirin, high-intensity statin, and maximally tolerated angiotensin-converting enzyme inhibitor on major adverse cardiac events in women with symptoms of ischemia but without obstructive epicardial CAD, a population with a high rate of CMD. In the population of patients with MINOCA, there is similarly the ongoing MINOCA-BAT trial examining the impact of beta blocker and ACEi/ARB therapy on adverse cardiovascular outcomes over 4 years.

### Anti-Anginal Treatment

Regarding anti-anginal therapy, beta blockers, nitrates, and calcium-channel blockers may have benefit in improving CMD [87–89] (Table 1). This is an evolving area of evidence, with recent work highlighting the importance of identifying and addressing the underlying pathophysiology for effective treatment.

In the Coronary Microvascular Angina (CorMicA) trial, 151 patients undergoing coronary angiography who were found to have no obstructive CAD were randomized to either have invasive coronary function testing that was used to guide their therapy or a sham coronary function testing (testing performed, but results not disclosed) [90••]. The coronary function testing results were divided into different endotypes: vasospastic angina, microvascular angina, both or neither. For patients randomized to coronary function testing with evidence of microvascular angina, consideration of aspirin, statin, and ACEi was recommended in all patients, and beta-blockers were recommended as first-line anti-anginal therapy. For patients with vasospastic angina (epicardial spasm), calcium channel blockers were recommended as first-line therapy. Patients whose therapy was guided by the invasive coronary function testing had improved angina scores and improved quality of life at 6 months compared to sham control [90••].

The recent Efficacy of Diltiazem to Improve Vasomotor Dysfunction (EDIT-CMD) trial was a randomized, placebo-controlled study of diltiazem, which assessed a coronary function test at baseline and again after 6 weeks of treatment [91]. Treatment with diltiazem reduced epicardial spasm compared to placebo, but did not improve other aspects of the coronary function testing and did not improve symptoms.

These results make a compelling case for the value of invasive coronary function testing, through which patients with vasospastic angina related to epicardial spasm can be distinguished from those with microvascular angina related to CMD, and thereby treated with medication targeting their specific disease process.

Multiple additional medications that primarily reduce angina have been studied in small, short-term pilot studies and demonstrated benefit with respect to symptom improvement, improved ischemia, and measures of CMD, including ranolazine [92, 93], ivabradine [94], trimetazidine [95], sildenafil [96], and nicorandil [94].

### Non-Pharmacologic Treatment

In addition to medications, non-pharmacologic therapies including smoking cessation, weight loss for obese patients, and aerobic training have been shown to improve invasive or non-invasive markers of CMD (Table 1) [89, 97–99].

## Conclusions

CMD occurs across a wide range of cardiovascular conditions, and is associated with a significant burden of symptoms and increased risk of adverse outcomes. There are powerful tools at the clinician’s disposal for invasive and non-invasive diagnosis of CMD, which assist in understanding the cause of symptoms and with prognostication. Recent work highlights the importance of stratified medical therapy guided by invasive coronary function testing to improve symptoms. Further research is needed to identify effective treatments to address the high rate of adverse outcomes related to CMD.
